# ﻿Karyotype diversity in the genus *Nysius* Dallas, 1852 (Hemiptera, Heteroptera, Lygaeidae) is much greater than you might think

**DOI:** 10.3897/compcytogen.17.116628

**Published:** 2023-12-18

**Authors:** Natalia V. Golub, Boris A. Anokhin, Valentina G. Kuznetsova

**Affiliations:** 1 Department of Karyosystematics, Zoological Institute, Russian Academy of Sciences, Universitetskaya emb. 1, 199034 St. Petersburg, Russia Department of Karyosystematics, Zoological Institute, Russian Academy of Sciences St. Petersburg Russia

**Keywords:** 18S rDNA, Ag-NOR, chromosome number, FISH, *Nysiusgraminicola*, Orsillinae, sex chromosomes, true bugs

## Abstract

We studied the karyotype and chromosomal distribution of 18S rDNA clustered in nucleolar organizer regions (NORs) in *Nysiusgraminicola* (Kolenati, 1845), belonging to the subfamily Orsillinae (Lygaeidae). It is shown that this species has a karyotype with 2n = 22(18+mm+XY), previously known in only one of 24 studied species of the genus *Nysius* Dallas, 1852, characterized by a similar karyotype, 2n = 14(12+mm+XY). In *N.graminicola*, 18S loci are located on sex chromosomes, which is a previously unknown trait for this genus. Our results in a compilation with previous data revealed dynamic evolution of rDNA distribution in *Nysius*. It is concluded that molecular chromosomal markers detected by FISH contribute to a better understanding of the structure and evolution of the taxonomically complex genus *Nysius*.

## ﻿Introduction

*Nysius* Dallas, 1852 is one of the most common and widely distributed genera within the family Lygaeidae (Heteroptera, Pentatomomorpha). Species of the genus are seed-predators; most species live in ruderal habitats and are often extremely abundant and sometimes becoming agricultural pests (Ge and Li 2019). The genus currently includes more than 100 described species and subspecies, with many more species remaining unrecognized (Ashlock 1967; Schaefer and Panizzi 2000; Péricart 2001; Nakatani 2015; Dellapé and Henry 2023). *Nysius* is a taxonomically complex group, and its members are known as “difficult to identify” because of the striking similarity of morphological features (Nakatani 2015). Obviously, some new methods and approaches are needed to solve the problem of distinguishing between closely related *Nysius* species. It has been shown that DNA sequencing of a standard gene region or ‘‘DNA barcoding’’ might speed a solution (Matsuura et al. 2012; Nakatani 2015).

Quite a few species of *Nysius* have been studied cytogenetically. Data on the number of chromosomes, the mechanism of sex chromosomes and, in some cases, the peculiarities of meiosis are currently available for 24 species, i.e. about 25% of all known species of this genus (reviewed by Ueshima and Ashlock 1980; see also Golub et al. 2023). Routine cytogenetics of *Nysius* appears to be highly conserved: all species have 2n = 14(12+XY), with the only exception being *N.tennellus* Barber, 1947, which has 2n = 22(20+XY). Each species has a pair of very small, so-called m-chromosomes (microchromosomes).

Consistent advances in chromosomal analysis increased dramatically in recent decades, becoming more refined and accurate through molecular cytogenetics using fluorescence *in situ* hybridization (FISH) allowing physical location of DNA sequences in chromosomes. The chromosomes of true bugs are holokinetic (Ueshima 1979), that is, they lack centromeres; therefore, the search for chromosomal markers is of great importance for the comparative analysis of their karyotypes. *rRNA* genes are among the better-known multigene families in true bugs (Panzera et al. 2021; Kuznetsova et al. 2021). The first recent application of FISH to map *rRNA* genes on the chromosomes of two *Nysius* species with modal karyotypes of 2n = 14(12+XY), *N.cymoides* (Spinola, 1837) and *N.helveticus* (Herrich-Schäffer, 1850), showed that they both have rDNA sites on the largest pair of autosomes (Golub et al. 2023).

The present study is focused on karyotype description of *N.graminicola* (Kolenati, 1845) based on classical cytogenetics, including Ag-NOR staining, and FISH mapping of the 18S rDNA probe, which, we believe, opens up new perspectives for understanding the evolution of karyotypes in the genus *Nysius*.

## ﻿Material and methods

Five males of *Nysiusgraminicola* were collected on August 15, 2023, 20 km NE of Voronezh (Russia) in a flood meadow on cereals. Males were freshly fixed in a mixture of alcohol and acetic acid (3:1) and stored in a refrigerator at 4 degrees until examination. Several slides were prepared from the testes of each male. Standard karyotypes were studied after staining by the Schiff–Giemsa method (Grozeva and Nokkala 1996). Nucleolus organizer regions (NORs) were localized by Ag-staining according to Howell and Black (1980) with minor modifications as described in Karagyan et al. (2020). To study the chromosomal distribution of major rDNA, FISH with an 18S rDNA probe of the firebug *Pyrrhocorisapterus* (Linneus, 1758) was performed according to the protocol described by Grozeva et al. (2015). The entire procedure (labeling, hybridizing, and detecting) is described in Golub et al. (2019) and Gokhman and Kuznetsova (2022). All preparations were photographed under oil-immersion (X100 objective) using a Leica DM 6000 B microscope, Leica DFC 345 FX camera, and Leica Application Suite 3.7 software with Image Overlay module (Leica Microsystems, Wetzlar, Germany). Filter sets A and L5 (Leica Microsystems) were used. The specimens from which chromosome preparations were made and the preparations themselves are stored at the Zoological Institute RAS (St. Petersburg, Russia).

## ﻿Results

### ﻿*Nysiusgraminicola* (Kolenati, 1845) n = 11 (9AA+mm+XY), 2n = 22, XY

**Figure 1.**
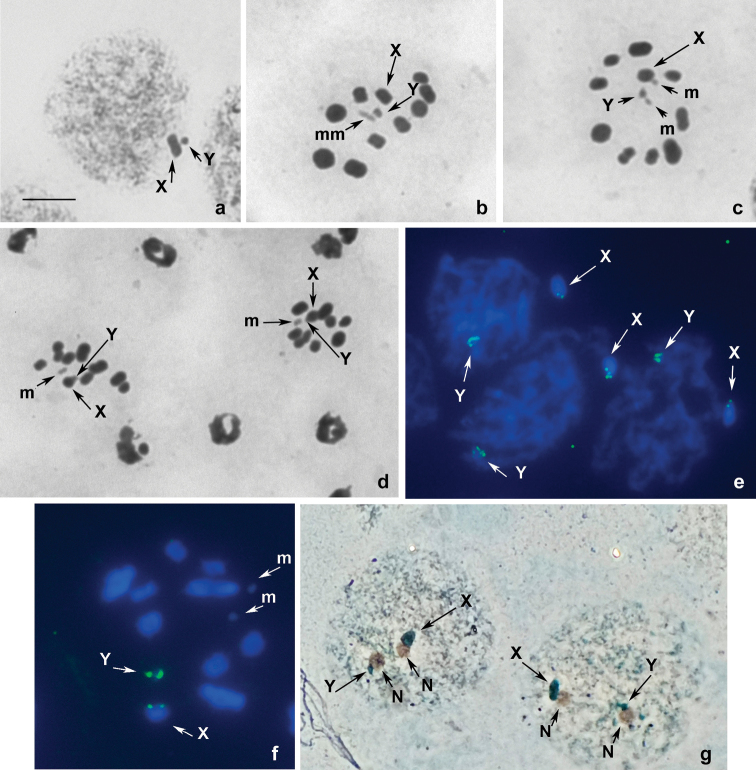
**a–g** Male meiotic karyotype of *N.graminicola* after standard staining (**a–d**), FISH with 18S rDNA probe (**e, f**), and Ag-staining (**g**) **a, e, g** interphase/prophase nuclei **b, c** metaphase I **f** diakinesis/MI transition **d** metaphases II, daughter cells. N – nucleolus. Scale bar: 10 µm.

The karyotype of *N.graminicola* has been studied for the first time. We analyzed the stages of male meiosis from prophase and metaphase I (MI) to metaphase II (MII) after the classic routine staining (Fig. 1a–d), after FISH with an 18S rDNA probe (Fig. 1e, f), and after Ag-staining (Fig. 1g). At the early prophase stages (Fig. 1a, e, g), there are two heteropycnotic bodies corresponding to the X-chromosome (presumably larger) and Y- chromosome (smaller); both lie on the periphery of the nucleus, sometimes far apart (Fig. 1e, g), but sometimes quite close to one another (Fig. 1a). At MI (Fig. 1b, c) and diakinesis/MI transition (Fig. 1f), there are 10 bivalents of autosomes, including a small pair of m-chromosomes, and sex chromosomes X and Y placed separately from each other. Eleven elements, including ten autosomes split into chromatids and a pseudobivalent XY, were found in each of the sister MII nuclei (Fig. 1d). It is obvious that sex chromosomes, unlike autosomes and m-chromosomes, segregate equationally in the first round of meiosis and divide reductionally in the second round of meiosis (inverted or post-reductional meiosis), which is characteristic of all Pentatomomorpha and most Heteroptera in general (Ueshima 1979). The meioformula of the karyotype of *N.graminicola* can thus be denoted as n = 9AA+mm+X+Y (2n = 22, XY). The autosomes form a decreasing size series; sex chromosomes, as noted above, are a different size and behave like univalents, each splitting into chromatids. M-chromosomes exhibit negative heteropycnosis during meiotic divisions; they may be located separately or form a pseudobivalent at prophase (not shown) and at MI (Fig. 1b, c), a phenomenon known as “touch-and-go” pairing studied in depth by Nokkala (1986) on the example of *Coreusmarginatus* (Linnaeus, 1758) (Coreidae). Both MI and MII plates are radial, with sex chromosomes and m- chromosomes lying in the center of a ring formed by bivalents (Fig. 1b, c, d). rDNA signals are visible on both sex chromosomes at all stages of meiosis, with larger and brighter signals on the Y-chromosome (Fig. 1e, f). Ag-staining revealed remnants of the nucleoli associated with both sex chromosomes in interphase/prophase cells, confirming the presence of *rRNA* genes in these chromosomes (Fig. 1g).

## ﻿Discussion

*Nysiusgraminicola* is the second species in the genus *Nysius* to have 2n = 22(20+XY). This karyotype was previously known only in *N.tennellus*, and its origin was attributed to autosome fragmentations in the karyotype with 2n = 14(12+XY), representing a plesiomorphic state common to vast majority of *Nysius* species (Ueshima and Ashlock 1980). It should be noted that this karyotype is one of two (second 2n = 16, XY) modal karyotypes in the family Lygaeidae including the subfamily Orsillinae (Ueshima and Ashlock 1980; Papeschi and Bressa 2006). The above hypothesis is confirmed by the fact that in the karyotype with 2n = 14 there is a pair of very large chromosomes (although for many species no karyotype illustration is given in the original publications), whereas in the karyotype with 2n = 22 (in both *N.graminicola* and *N.tennellus*) there is no such pair, and the chromosomes form a decreasing size series. The detection of a ribosomal cluster in autosomes in *N.cymoides* and *N.helveticus* sharing a modal karyotype (Golub et al. 2023) suggests an autosomal rDNA pattern to be the ancestral state for *Nysius*. Because the 18S ribosomal genes in these species are located on the largest pair of autosomes, we hypothesized that they would be found in one of the autosome pairs in *N.graminicola* with a derived karyotype. However, this hypothesis was not confirmed in our results, since the hybridization marks of the 18S rDNA probe were detected in the sex chromosomes of this species. Such a relocation of ribosomal sites from autosomes to the sex chromosomes is unlikely to be the result of chromosomal rearrangements alone. It is conceivable that transposable elements (also called “jumping genes” or mobile genetic elements) capable capturing entire genes and moving them from one genomic locus to another (Fambrini et al. 2020), could be involved in the dispersal of *rRNA* genes in the genus *Nysius*, as suggested for some other true bugs and some other insects (see examples and references in Panzera et al. 2021). The movement of rDNA clusters from autosomes to sex chromosomes is thought to be of evolutionary significance, causing genetic differentiation between divergent lineages and speciation events (see Pita et al. 2016; Panzera et al. 2021). We hypothesize that studies of other *Nysius* species will reveal a greater diversity of rDNA cluster distribution patterns, contributing to a better understanding of the structure and evolution of this taxonomically complex genus.

## ﻿Conclusion

Our results show that the genus *Nysius* is characterized by a much more pronounced karyotype diversity than previously thought.
